# Acute effects of resonance frequency breathing on cardiovascular regulation

**DOI:** 10.14814/phy2.14295

**Published:** 2019-11-28

**Authors:** Jeffrey Pagaduan, Sam SX Wu, Tatiana Kameneva, Elisabeth Lambert

**Affiliations:** ^1^ School of Health Sciences College of Health and Medicine University of Tasmania Tasmania Australia; ^2^ School of Health Sciences Department of Health and Medical Sciences Swinburne University of Technology Hawthorn Victoria Australia; ^3^ Faculty of Science, Engineering and Technology Swinburne University of Technology Hawthorn Victoria Australia; ^4^ Iverson Health Innovation Research Institute School of Health Sciences Faculty of Health, Arts and Design Swinburne University of Technology Hawthorn Victoria Australia; ^5^ Department of Biomedical Engineering University of Melbourne Parkville Australia

**Keywords:** baroreflex, blood pressure, resonance frequency, sympathetic nervous activity

## Abstract

Acute slow breathing may have beneficial effects on cardiovascular regulation by affecting hemodynamics and the autonomic nervous system. Whether breathing at the resonance frequency (RF), a breathing rate that maximizes heart rate oscillations, induces differential effects to that of slow breathing is unknown. We compared the acute effects of breathing at either RF and RF + 1 breaths per minute on muscle sympathetic nervous activity (MSNA) and baroreflex function. Ten healthy men underwent MSNA, blood pressure (BP), and heart rate (HR) recordings while breathing for 10 min at their spontaneous breathing (SB) rate followed by 10 min at both RF and RF + 1 randomly assigned and separated by a 10‐min recovery. Breathing at either RF or RF + 1 induced similar changes in HR and HR variability, with increased low frequency and decreased high frequency oscillations (*p* < .001 for both). Both respiration rates decreased MSNA (−5.6 and −7.3 bursts per min for RF and RF + 1 *p* < .05), with the sympathetic bursts occurring more often during mid‐inspiration to early expiration (+57% and + 80%) and longer periods of silence between bursts were seen (*p* < .05 for RF + 1). Systolic BP was decreased only during RF (−4.6 mmHg, *p* < .05) but the decrease did not differ to that seen during RF + 1 (−3.1 mmHg). The sympathetic baroreflex function remained unchanged at either breathing rates. The slope of the cardiac baroreflex function was unaltered but the cardiac baroreflex efficiency was improved during both RF and RF + 1. Acute breathing at either RF or RF + 1 has similar hemodynamic and sympatho‐inhibitory effects in healthy men.

## Abstract 2

Slow breathing at either a fixed pace or at the exact resonance frequency are strategies commonly used to lower blood pressure or improve symptoms such as anxiety. Whether breathing at the precise resonance frequency or simply at a low pace induce different acute effects on the autonomic nervous system is unknown. We evaluated the acute effects of the two types of breathing exercise in young healthy men on blood pressure, sympathetic nervous system activity, and baroreflex function. We find that regardless of the breathing scheme, slow breathing induced sympatho‐inhibition, decreased blood pressure and improved the efficacy of the cardiac baroreflex function. Our study demonstrate for the first time that breathing at the resonance frequency induces changes in the cardiovascular regulation similar to that of breathing at a slow pace.

## Graphical abstract

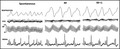

## 1 INTRODUCTION

Slow breathing has long been recognized to exert beneficial effects in a range of disorders including those related to the cardiovascular system and those associated with anxiety. Breathing at a low pace (5–6 breaths per min) has been shown to acutely decrease blood pressure (BP) in patients with post‐traumatic stress disorder (Fonkoue et al., 2018), and in the longer term decrease BP in patients with hypertension (Hering et al., 2013). In patients with heart failure, slow breathing either performed acutely or chronically reduced the cardiovascular reactivity to mental stress and improved various aspects of health‐related quality of life (Lachowska, Bellwon, Morys, Gruchala, & Hering 2019). Recently, an intervention has emerged consisting of breathing at a specific and individualized (slow) breathing rate, termed resonance frequency (RF), where oscillations in heart rate (HR) and breathing synchronize. This intervention typically involves the use of a device which guides an individual to breathe at their RF via visual or auditory feedback. Breathing at RF maximizes HR oscillations by creating a 0 degree phase shift between HR and respiration, while BP response from HR exhibit a 180 degree phase shift occurring at approximately 5‐s delay as a result of mechanical response (Vaschillo, Vaschillo, & Lehrer, 2006). Similar to slow breathing at a set pace, RF breathing has emerged as a promising tool to enhance performance, reduce stress and anxiety (Jester, Rozek, & McKelley, 2019; Lehrer & Gevirtz, 2014) and positively influence clinical symptoms in a number of disorders including depression (Lin et al., 2019), asthma (Taghizadeh, Eslaminejad, & Raoufy, 2019), and prehypertension (Lin et al., 2012).

The primary proposed mechanistic path for slow breathing or breathing at RF has been an improved vagal tone, associated with improved cardiac baroreflex function. Powerful within‐breath respiratory modulation of sympathetic vasoconstrictor activity has been well documented in humans (Macefield & Wallin, 1995; St Croix, Satoh, Morgan, Skatrud, & Dempsey, 1999). During spontaneous breathing, muscle sympathetic nerve activity (MSNA) is inhibited during mid‐inspiration to mid‐expiration, with MSNA activation occurring during late expiration (Eckberg, Nerhed, & Wallin, 1985; Limberg, Morgan, Schrage, & Dempsey, 2013). Interventions aiming at altering the breathing frequency may affect the within‐breath modulation of MSNA and hence modulate steady state sympathetic tone. Acute slow breathing for 10–15 min was found to decrease MSNA in patients with hypertension (Hering et al., 2013; Oneda, Ortega, Gusmao, Araujo, & Mion, 2010), post‐traumatic stress disorder (Fonkoue et al., 2018) and those with heart failure (Harada et al., 2014), but failed to induce any change in healthy subjects when the breathing exercise was of 3 min duration (Limberg et al., 2013).

Breathing at RF is thought to maximize heart rate variability (HRV) and influence autonomic nervous system (Lehrer & Gevirtz, 2014; Lehrer, Vaschillo, & Vaschillo, 2000; Lehrer et al., 2003); however, it is not known whether breathing at a precise respiration rate at an individualized RF may result in different changes in cardiovascular regulation compared to breathing at a set slow pace. The aim of the present study was therefore to compare the acute effects of breathing at an individual's RF relative to breathing at 1 breath above (RF + 1) on HR, HRV, BP, MSNA, within‐breath modulation of MSNA, and vascular and sympathetic baroreflex functions.

## 2 METHODS

### 2.1 Participants

Ten healthy non‐smoking males (age: 28.7 ± 4.8 years; height: 170 ± 7 cm; weight: 68.4 ± 8.0 kg) with no history of clinical disease volunteered in this study. Participants were required not to consume any food or any caffeine containing beverage for at least 2 hr before experimentation. The protocol of this study conformed to the Declaration of Helsinki for human experimentation. All subjects provided written informed consent prior to participation. The experimental protocol was approved by the Swinburne University Human Ethics Committee.

### 2.2 Procedures

This study involved two experimentation sessions separated by 24‐hrs. Determination of participant's RF was administered in the first session while breathing schemes were facilitated in the second session (HRV Starter System, Thought Technology). The RF was determined according to the protocol described by Lehrer and colleagues (2000) from incremental 2‐min paced breathing at various frequencies (6.5, 6.0, 5.5, 5.0, 4.5) while monitoring HR and respiration. At the end of 10 min, the breathing frequency that displayed the highest occurrence of low frequency in HRV (LF; 0.05–0.15 Hz) was selected as the RF. Prior to RF testing, participants underwent a biofeedback‐assisted paced breathing familiarization involving nasal inhalation and pursed‐lip exhalation.

On the second session, participants were placed in a semi‐supine position and instrumented for BP, HR, respiration, and MSNA recordings. After they had rested for at least 10–15 min, all parameters were continuously recorded for the ten minute baseline period where they were required to breathe at their spontaneous frequency. Following baseline recording, participants were asked to breathe at either their RF rate or RF + 1 for 10 min. Participants were allowed to rest for 10–15 min and told to breathe at their spontaneous rate until BP and MSNA values had returned to baseline before undergoing another 10‐min period of breathing at either their RF rate or RF + 1. Each participant therefore completed baseline first, followed by RF and RF + 1 in a random manner. During spontaneous breathing (SB), participants were asked to fix gaze at a blank computer screen. Paced breathing at RF or RF + 1 was guided by a visual pacer, with heart and respiratory oscillations displayed on screen.

### 2.3 Muscle sympathetic nerve activity, respiration, heart rate, and blood pressure

Recording of multiunit postganglionic MSNA was made with participants resting in a semi‐supine position as described previously (Lambert & Schlaich, 2004). A tungsten microelectrode (FHC) was inserted directly into the right peroneal nerve just below the fibular head. A subcutaneous reference electrode was positioned 2–3 cm away from the recording site. The nerve signal was amplified (350,000), filtered (bandpass 700–2,000 Hz), and integrated. During MSNA recording, BP was measured continuously using the Finometer system (Finapress Medical System BV), HR was determine using a lead III echocardiogram and respiration was assessed using a piezoelectric belt. BP, electrocardiogram, respiration, and MSNA were digitized with a sampling frequency of 1,000 Hz (PowerLab recording system, model ML 785/8SP; ADI Instruments).

### 2.4 MSNA analysis

Sympathetic bursts were visually identified and the number of bursts was averaged over the 10‐min period during SB, RF, and RF + 1. The MSNA was expressed as burst frequency (burst/min) and burst incidence (bursts/100 heartbeats). For analysis with simultaneous signals (BP, respiration), the MSNA signals were advanced 1.5 s to account for peripheral sympathetic nerve conduction delays (Fagius & Wallin, 1980). HR and BP were averaged over the 10‐min period during SB, RF, and RF + 1.

#### 2.4.1 Dynamic patterns of MSNA spiking

The inter‐burst interval was used to measure dynamic patterns of MSNA activity. The inter‐burst interval was used as a measure of burst‐suppression, when a cluster of bursts is followed by a silence period. The difference in inter‐burst intervals during each conditions was calculated for individual participants and then averaged for the whole cohort.

#### 2.4.2 Instantaneous phase of the respiration signal

To calculate the phase of the respiration signal, a Hilbert transform was applied to the signal. The Hilbert transform is a linear operator which converts a function of real variables into a complex plane (King, 2009). The instantaneous phase of the respiration is calculated as an arctangent of a ratio of real and imaginary parts of the signal, measured between −π and π, where π is the end of inspiration and early expiration and –π is the end of expiration and early inspiration. A histogram of MSNA burst occurrence was plotted as a function of an instantaneous phase of the respiration signal. The MSNA bursts histograms were normalized to the maximum value for each breathing period and compared among SB, RF, and RF + 1 breathing schemes.

### 2.5 Spontaneous arterial baroreflex control of MSNA

Over each period (SB, RF, RF + 1), diastolic BPs associated with individual heartbeats were grouped in intervals of 2 mmHg and, for each interval, the percentage of diastoles associated with a sympathetic burst was plotted against the mean of the pressure interval (Lambert et al., 2002). The sensitivity of the sympathetic baroreflex gain was defined as the slope of the regression line and was expressed as bursts/100 heartbeats/mmHg.

### 2.6 Spontaneous cardiac baroreflex sensitivity

Baroreflex sensitivity was assessed using the sequence method (Parati et al., 2004). This procedure identifies the ‘spontaneous’ sequences of three or more consecutive beats in which systolic BP progressively rose and cardiac interval progressively lengthened (type 1 sequences), or systolic BP progressively fell and cardiac interval progressively shortened (type 2 sequences), with a lag of one beat. For each sequence, the linear correlation coefficient between cardiac interval and systolic BP was computed and the sequence validated when *r* > 0.85. The slope between cardiac interval and systolic BP was calculated for each validated sequence and expressed as msec/mmHg. The baroreflex efficacy index (BEI) (Di Rienzo et al., 2001) was assessed as the total number of cardiac intervals/systolic BP sequences divided by the total number of systolic BP ramps.

### 2.7 Heart rate variability

HRV was assessed from the resting electrocardiogram (ECG) obtained during the MSNA recording and was determined using commercially available software (HRV Module for Chart 5 Pro; ADI Instruments, Bella Vista, Australia). Parameters derived were standard deviation of normal to normal intervals (SDNN) and standard deviation of heart rate (*SD* rate) in the time domain analysis. LF (0.04–0.15 Hz) and high frequency (HF: 0.15–0.4 Hz) in the frequency domain analysis expressed as percentage and normalized units. Additionally, LF/HF ratio was also included in HRV analysis.

### 2.8 Data analysis

In order to compare the variables under the three conditions (SB, RF, and RF + 1) a repeated measures ANOVA followed by pairwise multiple comparison procedure (Bonferroni *t*‐test) was used when the data were normally distributed. Otherwise, the Friedman's test for repeated measures was used and Wilcoxon signed rank test for paired samples was administered for post hoc analysis. Statistical analyses were carried out using a commercial statistical package (SPSS ver 25, IBM) with alpha set at 0.050 level. Data are presented as mean ± standard deviation or mean ± interquartile range.

## 3 RESULTS

**Figure 1:**
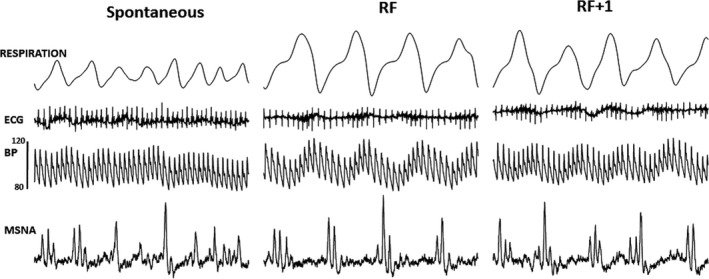
Original traces of respiration, ECG, blood pressure (BP), and muscle sympathetic nerve activity (MSNA) in one participant while breathing at their spontaneous breathing rate, at the resonance frequency (RF), and at the RF + 1. In this participant, MSNA is more pronounced during early inspiration and inhibited from mid‐inspiration to mid‐expiration with the effect being more obvious at RF and RF + 1

Figure 1 contains hemodynamic recordings from one subject showing the effects of breathing at the SB, RF, and RF + 1. Fluctuations in the ECG and BP signals are obvious at both RF and RF + 1. In this participant, MSNA was more pronounced during early inspiration, and subsequently inhibited from mid‐inspiration to mid‐expiration. This observation was more obvious at RF and RF + 1, compared with SB.

### 3.1 Respiration, blood pressure, and heart rate

As expected, the respiration rates during both RF and RF + 1 were significantly lower compared to that during SB (*p* < .01 for both) with RF + 1 being also significantly higher compared to RF (*p* < .01) (Table 1). Systolic BP was significantly lower during RF compared with SB (*p* < .05) but no difference was noted during RF + 1 (Table 1). No significant difference in either diastolic BP or HR were observed between breathing conditions.

**Table 1:** Hemodynamic parameters during spontaneous breathing (SB), breathing at the resonance frequency (RF) and at the RF + 1

	SB	RF	RF + 1
Hemodynamics			
Respiration rate, breaths/min	14.2 ± 3.13	5.44 ± 0.88**	6.43 ± 0.88^▲●^
Systolic blood pressure, mmHg	123.6 ± 12.13	119.0 ± 14.66*	120.5 ± 14.07
Diastolic blood pressure, mmHg	80.8 ± 11.8	78.7 ± 12.3	80.1 ± 11.5
Heart rate, bpm	71.2 ± 6.47	72.6 ± 7.73	72.5 ± 7.15

Note

Data are presented as mean ± *SD*.

*p < .050*, *RF versus SB, **p < .010, RF versus SB, *^▲^p < .*010, RF + 1 versus SB*,^ ●^p < .*010*,* RF versus RF + 1

### 3.2 Muscle sympathetic nervous activity

#### 3.2.1 Burst frequency and incidence

A significant difference in MSNA burst frequency and incidence was observed during both breathing schemes: Burst frequency during RF was significantly lower than SB (*p* < .05) (Table 2). Similarly, RF + 1 exhibited lower MSNA burst frequency than SB (*p* < .01). Likewise, lower burst incidence was seen during RF compared to SB (*p* < .05) and during RF + 1 compared to SB (*p* < .01). Difference in burst incidence and burst frequency between RF and RF + 1 was nonsignificant.

**Table 2:** Muscle sympathetic nerve activity (MSNA) and baroreflex function during spontaneous breathing (SB), breathing at the resonance frequency (RF), and at the RF + 1

	SB	RF	RF + 1
Muscle sympathetic nerve activity			
Burst frequency (Bursts per minute)	33.5 ± 9.67	27.9 ± 8.14*	26.1 ± 6.95*^▲▲^*
Burst incidence (Bursts per 100 heartbeats)	47.5 ± 15.0	38.9 ± 12.0*	36.5 ± 11.2*^▲▲^*
Burst occurrence as a function of respiratory phase [‐π,0]			
Normalized average burst occurrence	0.63 ± 0.09	0.44 ± 0.19	0.45 ± 0.22
Normalized median burst occurrence	0.65 (0.06)	0.43 (0.23)	0.46 (0.23)
Normalized maximum burst occurrence (corresponding phase)	0.82 (−π)	0.77(−π)	0.71(−π)
Difference in burst occurrence compared to SB at −π/2	–	−37%	−36%
Area under the curve of burst occurrence histogram	0.32 ± 0.07	0.36 ± 0.13	0.28 ± 0.08
Burst occurrence as a function of respiratory phase [0,π]			
Normalized average burst occurrence	0.40 ± 0.08	0.47 ± 0.15	0.56 ± 0.18
Normalized median burst occurrence	0.41(0.10)	0.46 (0.17)	0.58 (0.30)
Normalized maximum burst occurrence (corresponding phase)	0.49(π)	0.62(π/2)	0.72(π/2)
Difference in burst occurrence compared to SB at π/2	–	+57%	+80%
Area under the curve of burst histogram	0.15 ± 0.05	0.28 ± 0.14*	0.33 ± 0.13*^▲▲^*
Inter bursts intervals corresponding to < 4 ms			
Average	0.41 ± 0.09	0.41 ± 0.12	0.44 ± 0.13
Median	0.38 (0.12)	0.40 (0.12)	0.47 (0.18)
Maximum (corresponding interval)	1 (1.25 ms)	1 (1.25 ms)	1 (1.25 ms)
Difference compared to SB	–	+0.02	+0.13
Area under the curve of inter burst interval	1.12 ± 0.08	1.11 ± 0.12	1.07 ± 0.11
Inter bursts intervals corresponding to ≥ 4 ms			
Average	0.02 ± 0.02	0.03 ± 0.03	0.05 ± 0.04*^▲^*
Median	0.01(0.02)	0.02 (0.04)	0.04 (0.04)
Maximum (corresponding interval)	0.09 (5 ms)	0.27 (5 ms)	0.19 (5 ms)
Difference compared to SB	–	+0.13	+0.26
Area under the curve of inter burst interval histogram	0.12 ± 0.09	0.16 ± 0.11	0.19 ± 0.09*^▲^*
Sympathetic baroreflex function slope, bursts/100 heartbeats/mmHg	−2.92 (2.55)	−4.07 (3.27)	−4.04 (4.19)
Cardiac baroreflex function slope, msec/mmHg	14.6 ± 3.83	13.8 ± 4.51	14.1 ± 5.51
Cardiac baroreflex function efficacy index	47.3 (30.5)	52.9 (33.1)*	61.3 (20.1)^▲^

Note

Data are presented as mean ± *SD*. *p < .050, RF versus SB, ^▲^ *p* < .050, RF + 1 versus SB, ^▲▲^ *p* < .010, RF + 1 versus SB. Data are presented as mean ± *SD* or median (interquartile range).

#### 3.2.2 Pattern of sympathetic activity

**Figure 2:**
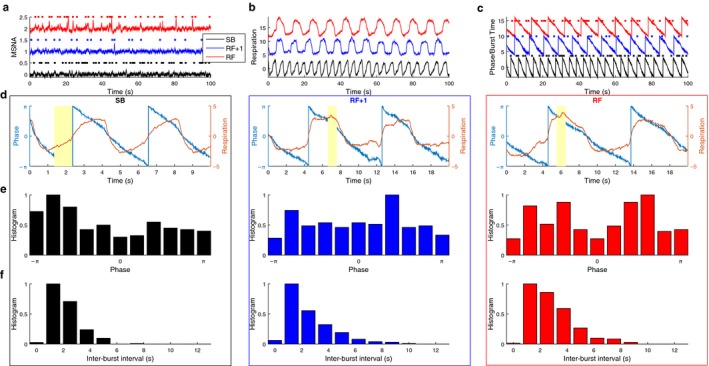
Examples of recorded and analyzed data from a participant. (a–d) illustrate short extracts of data for an individual participant. (e and f) illustrate data for the whole period of recording for the same participant. All three conditions are shown in subplots (a–b), shifted along vertical axis for clarity, and color‐coded: Black—spontaneous breathing (SB), Red—resonance frequency (RF), Blue—at (RF + 1). (a) An extract of muscle sympathetic nerve activity (MSNA) data (solid lines) and detected bursts (stars). (b) Respiration signal for three conditions. (c) Detected phase of respiration signal from b (solid lines) and MSNA bursts from a (stars), aligned in time. (d) An extract of respiration data (left y‐axis) and detected phase of the signal (right y‐axis). Yellow rectangles: the phase of the signal when maximum likelihood of spiking occurs. (e) Normalized burst time histogram as a function of the instantaneous phase of respiration. (f) Normalized histogram of inter‐burst intervals. (d–f) Data for baseline SB (left plot), RF + 1 (middle), and RF (right)

Figure 2 shows an example of MSNA data with corresponding respiration and the calculated instantaneous phase of the signal, normalized histogram of burst occurrence as a function of instantaneous phase of the respiration signal and normalized inter‐burst interval histogram.

#### 3.2.3 Burst histogram as a function of respiration phase

**Figure 3:**
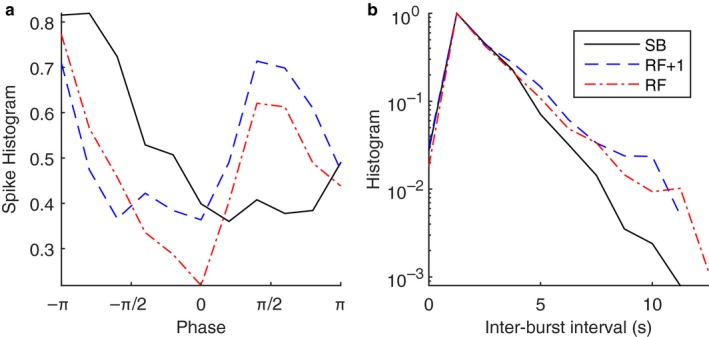
(a) Burst occurrence as a function of respiration phase averaged between all participants and normalized to a maximum value. There is a significant increase in burst occurrence on the 0 to π interval during breathing at resonance frequency (RF) and at the RF + 1. At this interval, the maximum likelihood of burst occurs at π phase for a baseline condition (spontaneous breathing, SB), while for the RF and at the RF + 1 the maximum occurrence of burst is seen at −π/2. On average, there is a 68% increase in burst occurrence at the π/2 phase during RF and at the RF + 1 compared to the SB. (b) inter‐bursts interval data averaged between all participants. It can be seen that inter‐burst intervals become longer during RF and at the RF + 1 breathing patterns, that is, there are more silence periods

The average, median, maximum, and differences in MSNA burst occurrence for the phase intervals −π to 0 and 0 to π are presented in Table 2 and the average across all phases is illustrated in Figure 3a. For the phase intervals −π to 0, no significant difference existed between breathing frequencies. For the phase interval 0 to π, there was a difference in the area under the curve of burst occurrence histogram from 0 to π with both RF and RF + 1 displaying significantly greater area under the curve of burst occurrence histogram compared to SB (*p* < .05 and *p* < .01, respectively).

#### 3.2.4 Inter‐burst intervals

Inter‐burst intervals were divided in duration of <4 ms and ≥4 ms. The mean and maximum inter‐burst intervals for each duration are presented in Table 2 and Figure 3b illustrates inter‐burst interval data averaged between all participants*.* There was no difference between breathing schemes in inter‐burst intervals for the <4 ms. However, there was a significant difference in inter‐burst intervals at ≥4 ms with RF + 1 displaying a higher inter‐burst intervals compared to SB (*p* < .05). The area under the curve of inter‐burst intervals ≥4 ms was larger during RF + 1 compared to SB (*p* < .05). Similar trends were noticed during RF but these did not reach significance.

### 3.3 Baroreflex function

The slopes of both sympathetic and cardiac baroreflex functions were unchanged during breathing exercises. The BEI of the cardiac baroreflex function was significantly increased during RF and RF + 1 (*p* < .05 for both).

### 3.4 Heart rate variability

All changes in HRV for time domain and frequency domain analysis are presented in Table 3. In the time domain *SD* rate was higher during RF and RF + 1 (*p* < .05 and *p* < .01). In the frequency domain, RF and RF + 1 displayed greater LF Power and lower HF power compared to SB but no significant difference were found between RF and RF + 1.

**Table 3:** Heart rate variability parameters during spontaneous breathing (SB), breathing at the resonance frequency (RF), and at the RF + 1

	SB	RF	RF + 1
Time domain analysis			
*SD* rate	5.10 ± 1.45	7.30 ± 2.41*	7.20 ± 2.30^▲▲^
SDNN ms	48.0 ± 25.4	46.2 ± 26.9	52.3 ± 28.2
Frequency domain analysis			
LF power (%)	28.0 ± 12.6	69.6 ± 13.2**	64.6 ± 19.1^▲▲^
LF power (nu)	46.8 ± 20.8	88.3 ± 6.11**	82.1 ± 12.4^▲▲^
HF power (%)	32.2 ± 16.3	9.30 ± 6.03**	12.8 ± 8.90^▲^
HF power (nu)	51.2 ± 19.5	11.5 ± 5.76**	17.2 ± 11.8^▲^
LF/HF	1.60 ± 2.22	10.9 ± 8.47**	8.90 ± 7.55^▲^

Note

Data are presented as mean ± *SD*. *p < .050, RF versus SB, ^▲^ *p* < .050, RF + 1 versus SB, ^▲▲^ *p* < .010, RF + 1 versus SB.

Abbreviations: HF, High frequency; LF, Low frequency;* SD*, Standard deviation; SDNN, standard deviation of normal to normal intervals.

## 4 DISCUSSION

This study examined the effects of short term breathing at RF on cardiovascular regulation compared to breathing at 1 breath/min above the RF. We addressed this issue by assessing hemodynamics including direct sympathetic nerve recording and assessment of cardiac and arterial baroreflex function. Major findings are: (a) Both RF and RF + 1 induced similar changes in MSNA including a decrease in the incidence and frequency of bursts, with the bursting pattern associated with longer periods of burst silencing and a shift of burst occurrence towards mid‐inspiration to early expiration, (b) RF and RF + 1 were both associated with improved cardiac baroreflex efficacy but did not affect the sympathetic baroreflex function (c) RF induced a significant reduction in systolic BP.

This is the first study to examine the modulatory effects of respiration at RF and RF + 1 on sympathetic activity investigating both the global sympathetic tone (burst incidence and frequency) and the dynamic pattern of sympathetic firing including inter‐burst intervals and burst occurrence as a function of the respiration phase. A significant reduction in sympathetic burst frequency and incidence were seen during either RF or RF + 1 compared with SB. In the healthy state, MSNA represents global sympathetic outflow to the skeletal muscle linked to BP regulation with strong feedback from the arterial baroreceptors (Wallin, 2006) and respiratory modulation (Habler, Janig, & Michaelis, 1994). The reduction in MSNA observed during slow breathing at either RF or RF + 1 was similar to that demonstrated in previous studies where slow breathing was of 10–15 min duration (Fonkoue et al., 2018; Harada et al., 2014; Hering et al., 2013; Oneda et al., 2010). Two prior studies in healthy subjects indicated no effect of slow breathing on MSNA but the duration of the breathing exercise was much shorter being 3 (Limberg et al., 2013) or 4 min (Raupach et al., 2008). Our data indicate that MSNA is reduced during slow breathing in healthy subjects with a longer breathing task. Lower burst frequency and burst incidence with RF or slow breathing in general may be due to activation of pulmonary mechanoreceptors in response to the increased tidal volume that accompanies slow breathing. This is supported by the findings of Lehrer & colleagues (2003) who demonstrated greater tidal volume among healthy male participants under RF breathing compared with SB. The increased tidal volume under slow breathing might have activated lung stretch receptors and reduced chemoreflex response thus suppressing the activation of MSNA (Bernardi, Gabutti, Porta, & Spicuzza, 2001; Seals, Suwarno, & Dempsey, 1990). The modulatory influence of breathing on MSNA has previously been described with approximately 70% of the activity occurring during low lung volumes (initial half of inspiration and latter half of expiration) and MSNA decreasing progressively and markedly from onset to late inspiration (Seals et al., 1993). The analysis of the pattern of sympathetic activity using the respiration phase analysis and the inter‐burst intervals is novel and allows to demonstrate that RF and RF + 1 have significant effect on the pattern of sympathetic activity. In accordance to that described by Seals et al. (1993) we documented that during normal spontaneous breathing, MSNA is more likely to occur during the −π to −π/2 phase indicating higher activity during early inspiration and late expiration. MSNA is more inhibited during the 0 to π phase, hence during the late inspiration to early expiration. The decrease in burst incidence and frequency observed during either RF or RF + 1 is associated with a change in the pattern of bursting with the bursts being less frequent during the respiration phase interval −π/2 to 0 (expiration to onset of inspiration) and more frequent around π/2 phase (late inspiration). In addition, the inter‐burst interval analysis revealed longer periods of burst silencing during RF and RF + 1. Hence, the sympatho‐inhibition observed during either RF or RF + 1 seems to occur as a result of changes in the bursting pattern of MSNA imposed by the ability of the respiration to modulate the timing of bursts. Such changes are potentially critical because the respiratory‐modulated bursting of sympathetic activity has been shown to modulate vascular resistance (Briant, O'Callaghan, Champneys, & Paton, 2015).

Slow breathing exercises have been reported to decrease BP albeit to a modest extent (Fonkoue et al., 2018; Grossman, Grossman, Schein, Zimlichman, & Gavish, 2001; Hering et al., 2013; Rosenthal, Alter, Peleg, & Gavish, 2001). In line with these previous studies, we noticed a small but significant decrease in systolic BP during the RF breathing with no difference in systolic BP between RF and RF + 1 indicating that breathing at RF as opposed to RF + 1 did not trigger a better systolic BP response. Breathing at RF has been proposed to modulate autonomic cardiovascular regulation, affecting cardiopulmonary reflexes, arterial baroreflexes, sympathetic vascular tone, and peripheral resistance, which in turn may result in systemic vasodilatation and decreased BP (Lehrer & Gevirtz, 2014).

Enhanced baroreflex sensitivity has been suggested to occur during slow breathing (Bernardi et al., 2001; Raupach et al., 2008) or breathing at RF (Lehrer et al., 2000), possibly as a result of reduced chemoreflex sensitivity, which in turn may lead to decreased sympathetic tone. Our study indicates that the slope of the cardiac baroreflex function (assessed as a combination of up and down sequences) remains unchanged during RF or RF + 1 but the efficacy of the baroreflex as assessed by BEI was improved during both RF + 1 and RF. BEI has been suggested to be a good representation of the baroreflex function in healthy subjects as it quantifies the number of times the baroreflex is effective in driving the sinus node (Di Rienzo et al., 2001). Tzeng et al. (2009) demonstrated that slow breathing in healthy subjects did not affect the arterial baroreflex when measured using the gold standard modified Oxford method and suggested that results indicating improvement in baroreflex function during slow breathing may have occurred as commonly used baroreflex assessment techniques may not be accurate in this setting. The sympathetic baroreflex function was also explored as a possible contributor to the changes in MSNA as Fonkue and colleagues observed that slow breathing improved the sympathetic baroreflex function (Fonkoue et al., 2018). However, this was not observed in our study as neither RF nor RF + 1 improved the slope of the sympathetic baroreflex function. Overall, the effects of slow breathing schemes in this study on baroreflex function are still unclear as results vary depending on the method used (Tzeng et al., 2009).

Within the context of this study, we found that breathing at RF or RF + 1 induced significant hemodynamic and autonomic changes but we were unable to detect any differences between the two breathing schemes. This raises the question as to whether precise measurement of the RF is essential for the reported beneficial clinical effects of individualized RF or a standardized paced breathing at 5–7 breaths per min is all that is required. Lin et al. (Lin et al., 2012) investigated the effects or either paced breathing at RF compared to slow breathing (6 breaths/min) in individuals with prehypertension and found that both schemes of breathing resulted in a decrease in BP over a period of 5 weeks and that the decrease in BP was more marked in those breathing at RF except for the first session where the BP fall was identical with either RF or RF + 1. They also found that RF induced stronger changes in HRV indices and in the baroreflex function compared with slow breathing which may explain better hemodynamic changes. In this study, we noticed that there was neglible difference in HRV and the respiratory modulation of MSNA between acute RF and RF + 1. These findings indicate occurence of sympathoinhibition regardless of RF or RF + 1. In a recent study comparing RF and RF + 1 on BP levels, Steffen et al. (Steffen, Austin, DeBarros, & Brown, 2017) noticed that both RF and RF + 1 decreased systolic BP to the same extent but subjects allocated to the RF exercise exhibited lower systolic BP in response to a stress test compared to subjects allocated to RF + 1. This was accompanied by better mood suggesting a preferential effect of RF compared to RF + 1 on the stress response. Hering et al. (Hering et al., 2013) showed that slow breathing at 6 breaths per min induced significant decrease in BP in the long term but not acutely in hypertensive subjects. Interestingly, they found, that long‐term paced breathing selectively attenuated pressor and tachycardic responses to mental stress but the corresponding MSNA responses remained unaltered; however, in their study the resonance frequency was not imposed. Hence, while slow breathing in general is undoubtedly a strategy to improve BP and stress responses, whether breathing at the specific RF is more efficient in the longer term is uncertain. More studies are certainly needed to fully examine long‐term effect of breathing at RF on hemodynamic markers.

Limitations in the current study should be acknowledged. This study was conducted in a small group of healthy young males. Hemodynamic and MSNA results in this cohort might not be applicable in other populations. Slow breathing may be more beneficial in individuals with elevated sympathetic nervous activity and decreased baroreflex function such as those with hypertension, heart failure, chronic obstructive pulmonary diseases, or in individuals with anxiety disorders where sympathetic and vagal function may be altered. Also, this study was conducted in a single session and respiratory rate was assessed using a standard respiratory belt hence parameters such as tidal volume were not available. Future studies should employ long‐term applications of RF versus RF + 1 to identify dose–response relationship on hemodynamic and autonomic nervous system.

## 5 CONCLUSION

Breathing exercises remain an attractive non‐invasive strategy to modulate autonomic nervous system function. While breathing at the specific RF is suggested to maximize the effect, we found that breathing at RF or 1 breath above RF induced the same acute changes on the sympathetic nervous system and BP, with both breathing paradigms inducing similar changes in the pattern of sympathetic firing.

## CONFLICT OF INTEREST

None declared.
